# A Survey on AI-Driven Mouse Behavior Analysis Applications and Solutions

**DOI:** 10.3390/bioengineering11111121

**Published:** 2024-11-06

**Authors:** Chaopeng Guo, Yuming Chen, Chengxia Ma, Shuang Hao, Jie Song

**Affiliations:** 1 Software College, Northeastern University, Shenyang 110169, China; guochaopeng@swc.neu.edu.cn (C.G.); 2290126@stu.neu.edu.cn (Y.C.); 2 College of Life and Health Sciences, Northeastern University, Shenyang 110169, China; 2101365@stu.neu.edu.cn

**Keywords:** mice behavior analysis, mice model, AI, computer vision

## Abstract

The physiological similarities between mice and humans make them vital animal models in biological and medical research. This paper explores the application of artificial intelligence (AI) in analyzing mice behavior, emphasizing AI’s potential to identify and classify these behaviors. Traditional methods struggle to capture subtle behavioral features, whereas AI can automatically extract quantitative features from large datasets. Consequently, this study aims to leverage AI to enhance the efficiency and accuracy of mice behavior analysis. The paper reviews various applications of mice behavior analysis, categorizes deep learning tasks based on an AI pyramid, and summarizes AI methods for addressing these tasks. The findings indicate that AI technologies are increasingly applied in mice behavior analysis, including disease detection, assessment of external stimuli effects, social behavior analysis, and neurobehavioral assessment. The selection of AI methods is crucial and must align with specific applications. Despite AI’s promising potential in mice behavior analysis, challenges such as insufficient datasets and benchmarks remain. Furthermore, there is a need for a more integrated AI platform, along with standardized datasets and benchmarks, to support these analyses and further advance AI-driven mice behavior analysis.

## 1. Introduction

Mice are one of the animal models in the biology and medical fields. This technology has been utilized for many years. It offers several advantages, such as its similarity to humans in various physiological functions and the ability to perform functional interventions through genetic modification. Researchers conducted various experiments on mice and observed the experimental phenomena of mice for biological and medical study, such as gene identification [1], cell classification [2] and protein prediction [3]. Among the in vivo and in vitro experiments, mice behavior analysis is an essential topic and plays critical roles in medicine, neuroscience, biology, genetics, and educational psychology. For instance, scientists examine the behavioral patterns of mice to explore the impact of gene mutations, assess the effectiveness of potential drug therapies, or reveal the neural mechanisms underlying behavior to advance the treatment of mental disorders. Today, analyzing mouse behavior is prevalent across various biomedical research areas.

In the initial phases of research, conventional behavioral analysis methods enable the quantification of animal behavior by tracking their spatial position using techniques like the three-chamber assay [4], open-field test [5], and water maze [6]. However, as technology advances, these traditional methods face challenges in capturing critical details of behavior that involve subtle movements [7]. Fine-grained behavioral features cannot be accurately captured through visual observation or subjective assessment alone. Additionally, traditional methods are often time-intensive when computing high-precision features, and the results can vary significantly [8]. A novel, automated, quantifiable approach for extracting fine-grained behavioral features is essential.

With the advancement of artificial intelligence, AI can learn from large amounts of data and extract quantitative features automatically. “AI+” has become a research and application trend in many fields today, including medical engineering analysis and biological [9]. AI models have been employed in the medical field to enhance diagnostic accuracy, predict disease progression, and optimize treatment plans. For instance, deep learning methods have been used to automatically analyze medical images, allowing for early detection of conditions such as cancer or cardiovascular diseases. In the biological sciences, AI analyzes large data sets, such as genome sequences, to identify underlying patterns and make predictions. Similarly, AI has seen a surge in applications in animal behavior analysis. Traditional methods of behavior analysis, such as manual observation or basic motion tracking, are being replaced by AI-powered approaches that offer more accuracy, speed, and scalability. AI enables the automatic extraction of high-dimensional behavioral features from video data. These advanced methods have been applied to tasks like recognizing specific movements, detecting abnormal behaviors, and identifying previously undetectable patterns in the human eye. Specifically, in mice behavior analysis, researchers have also integrated AI into the analysis of mouse behavior by examining video or video frame data using techniques like machine learning [10] and deep learning [11]. AI enhances the analysis of mouse behavior, enabling innovative research that was previously unattainable.

In recent years, AI techniques have been advancing rapidly, and AI-empowered studies on mice behavior analysis also become popular. Various AI approaches are applied in various types of mice behavior analysis applications, and AI approaches have relevance with the applications, which can refer to researchers and mine more potential AI-empowered solutions for the same mice behavior analysis applications. Besides, the applications can be categorized into several typical AI tasks. Each AI task has various AI approaches corresponding to a mouse behavior analysis application with several patterns. Therefore, the applications, AI tasks, and AI approaches are tightly connected. It is valuable to make a study to conclude the relationship among them as a reference for biology-related researchers. This paper mainly surveys the AI-empowered studies on mice behavior analysis and summarizes the relationship among their applications, AI tasks, and AI approaches. Concretely, this paper starts the survey with three research questions (RQ):

- RQ1: What applications can AI empower in the mice behavior analysis studies? (Answered in Section 3)
- RQ2: How to taxonomize the applications into AI tasks? (Answered in Section 3)
- RQ3: What methods are commonly used in artificial intelligence tasks related to mouse behavior analysis? (Answered in Section 4)

The rest of the paper is structured as follows: Section 2 summarizes the survey method of this paper, including the survey route and literature searching. Section 3 summarizes all the applications on the mice behavior analysis and proposes the relationship between applications and AI tasks. Section 4 summarizes the suitable AI-empowered task approaches. Section 5 discusses the limitations of artificial intelligence technology in mouse behavior analysis and the advantages and challenges of constructing a mouse behavior analysis platform framework. Section 6 concludes the paper.

## 2. Survey Method

This section summarizes the survey method used in this paper, including survey route and literature searching.

For the survey route, this paper aims to survey the studies of AI-empowered mice behavior analysis. We first search the studies, categorize studies into applications, and summarize each study’s AI tasks. Then, we introduce the AI tasks that appeared in studies and summarize several state-of-the-art AI approaches for each AI task. Lastly, we combine the applications, AI tasks, and AI approaches into an LLM system architecture.

For the literature search, we conducted a preliminary search about mice behavior with AI approaches using the Google Scholar and SCI Expanded library with the keywords “mice behavior AND machine learning AND deep learning”. In Google Scholar, the keywords are chiefly matched in the body of papers instead of the abstract, and the search results contain the patents and research reports. They are not our primary focus. In the SCI Expanded library, we search the exact keywords in the title, abstract, and keywords. The search scope is “Article AND Meetings”. The initial number of retrieved documents amounted to around 85 publications.

The paper selection process is a critical step to ensure the selected literature’s relevance and quality. To enhance the objectivity and reliability of the selection process, we implemented the following measures:

Involvement of Multiple Researchers: The selection process involved multiple researchers to reduce the impact of individual biases. Specifically, three researchers participated in the screening and evaluation of the literature.

Establishment of Selection Criteria: Before the selection began, the research team jointly established clear selection criteria. These criteria included data type (such as video or video frames), research objectives (specific application goals rather than technical goals), and research field (machine learning domain). These standards provided objective guidance for the selection process. The specific inclusion and exclusion criteria are as follows:

- Including studies whose data are videos or video frames;
- Including studies that have exact application goals instead of technical goals;
- Including studies in the field of machine learning;
- Excluding studies that applied methods originally developed for human behavior analysis without adaptation for mice.
- Excluding studies whose data are not videos or video frames;
- Excluding studies that only have technical goals instead of specific application goals;
- Excluding studies that are not in the field of machine learning.

Independent Evaluation and Cross-Validation: Each researcher independently evaluated the literature and scored it based on the pre-established criteria. Subsequently, the research team combined their evaluation results and conducted cross-validation to ensure consistency.

Discrepancy Resolution Mechanism: For any discrepancies that arose during the evaluation process, the research team resolved them through discussion and negotiation. Specific methods included holding group meetings to discuss the strengths and weaknesses of each paper in detail and re-evaluating them based on the selection criteria. If necessary, the team would bring in additional researchers for third-party evaluation to ensure the fairness of the final decision.

**Figure 1:**
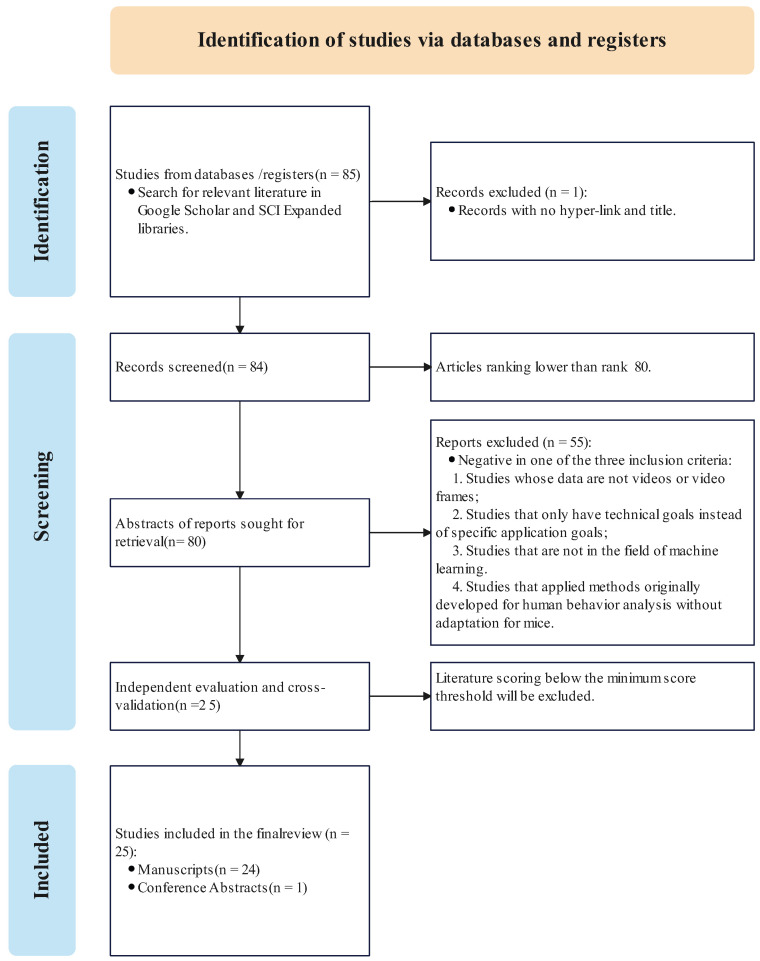
The overall process of screening relevant literature.

Our study ranks the retrieved relevant literature by relevance and selects the top 80 studies as candidates. Subsequently, three researchers screen these studies based on the established selection criteria and independently evaluate them, assigning scores to each piece of literature and excluding those below the threshold. If discrepancies arose during the evaluation process, the subsequent steps were executed according to the discrepancy resolution mechanism. Ultimately, we selected 25 papers as state-of-the-art works based on the process above. Figure 1 shows the screening process of relevant literature.

## 3. Applications

This section summarizes the state-of-the-art AI-empowered mice behavior research applications to summarize and taxonomize AI-empowered mice behavior analysis applications to study the mice behavior analysis platform framework further.

### 3.1. Disease Detection

**Figure 2:**
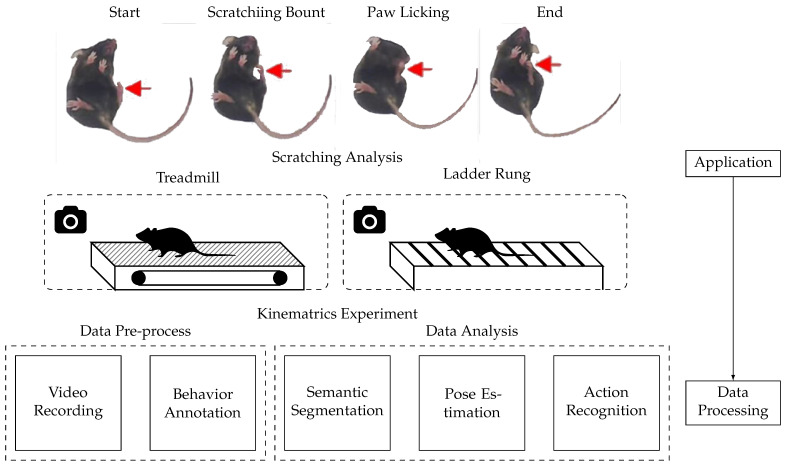
Example of Artificial Intelligence Diagnosis of Mouse Diseases.

Changes in daily human behavior (e.g., food intake, sleep, and activity patterns) can often reflect symptoms of several diseases. Mice disease models [12,13] are a valuable resource in studying the diseases [14]. However, these studies require long and systematic observations of disease-carrying mice, which requires much labor and is subject to human error. Fortunately, AI can be a powerful tool for diagnosing disease in mice [15,16,17]. As shown in Figure 2, mice behaviors, such as scratching and gait, are recorded as video data with high-speed cameras. AI methods, such as semantic segmentation, pose estimation, and action recognition, diagnose disease in mice through video data. AI offers fresh perspectives on the pathophysiology and treatment of various diseases.

Most existing studies on AI-empowered mice disease detection are based on video data, with a few using text data. Yu et al. [18] record mice behavior from the bottom of a videotaping box, effectively capturing essential body parts involved in scratching. Weber et al. [19] design a custom runway with mirrors for 3D recording from side and bottom views, allowing detailed gait analysis and refinement of the ladder rung test. Their deep learning approach accurately tracks recovery in rodents post-stroke. Yu et al. [18] developed Scratch AID, a system using a Convolutional Recurrent Neural Network (CRNN) for high-accuracy scratch detection, achieving a recall of 97.6% and a precision of 96.9%. Sakamoto et al. [16] create a similar method for black mice, using CRNN and a posterior filter to improve prediction accuracy by filtering out short sequences prone to errors. Aljovic et al. [20] introduce an open-source toolbox for neurological conditions featuring pose estimation and image classification to compute kinematic parameters and detect footfalls. This comprehensive analysis identifies locomotor function parameters optimal for tracking brain or spinal cord injuries. Alexandrov et al. [10] use Support Vector Machines to analyze phenotypic data, identifying phenotypes that differentiate mice with varying CAG repeat lengths. Their model accurately predicts CAG-repeat length, highlighting the potential to predict disease mutations through subtle behavioral phenotype variations.

### 3.2. External Stimuli Effective Assessment

**Figure 3:**
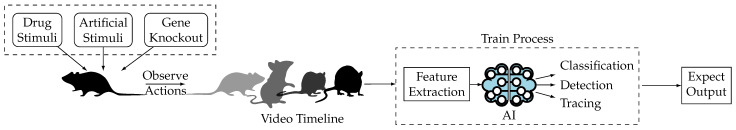
Examples of Artificial Intelligence Automated Assessment of Small External Stimulus Effects.

Effective assessment of external stimuli is a basic experiment approach for mice. Compared with mice without stimuli, researchers use external stimuli on specific mice organs to analyze the stimuli effect by analyzing mice behaviors. The types of external stimuli are various, such as medicine [21], artificial stimulus [22], and genetic alteration [23]. Due to the high-speed behavior of mice, traditional approaches cannot exactly obtain the video frames with the complete organ. With the development of AI technology, researchers apply AI-empowered approaches to evaluate the external stimuli’ effect on mice automatically, which presents the basic research steps in Figure 3. Researchers make various external stimuli on the mice, such as drug stimuli, artificial stimuli, and gene knockout, to observe the actions of mice. With the AI techniques empowering, the AI models extract the features from the video timeline and make classification, detection, and tracking tasks through the training process. Then, the researchers can get the expected outputs from the AI models. Studying the effects of external stimuli on mice can contribute to exploring disease treatment and neuroscience. They generally focus on the detection, classification, segmentation, and tracking tasks.

Current AI-empowered studies on effective assessment of external stimuli adopt video data as the training and testing data. Wotton et al. [24] collect video data to analyze mice behavior in response to a hind paw formalin injection. Kathote et al. [25] record the bottom view videos of the mice’s behavior with acetazolamide and baclofen. Vidal et al. [26] create a video database including the behavioral data of 8 white-haired mice collected multiple times. Abdus-Saboor et al. [27] utilize high-speed videography to capture sub-second, full-body movement videos. Marks et al. [28] gather raw video frames directly in complex environments. Torabi et al. [29] collect video recordings of neonatal (10-days-old) rat pups using standard locomotor-derived kinematic measures. Martins et al. [30] collect videos of the tail suspension test in a controlled environment. Wang et al. [31] collect mice behavior with an overhead camera during video recording.

The studies of AI-empowered external stimuli’ effective assessment can be categorized into medical stimulus, artificial stimulus, and genetic alteration. Wotton et al. [24] proposed an automated scoring system for the formalin assay using key point detection with DeepLabCut [32] and a GentleBoost classifier to identify licking behavior. Results showed that the system efficiently scored over 80 videos, revealing strain differences in response timing and amplitude. Vidal et al. [26] automated grimace scale prediction in mice using AI for detection and classification. YOLO and a Dilated CNN segmented eye region were used to detect stable frame views. The method differentiated various pain scales effectively. Abdus-Saboor et al. [27] used AI-driven action recognition to analyze behavioral features post-stimulation, using machine learning to classify pain-like probabilities. This sensitive pain assessment utilized behavioral calibration. Kathote et al. [25] developed an AI pose estimation method to quantify behaviors in glucose transporter one deficiency syndrome mice, indicating potential therapeutic insights for cancer. The method effectively estimated preclinical suitability. Marks et al. [28] introduced a deep learning architecture to study brain function through behavior quantification, successfully recognizing 3D behaviors in mice and primates. Torabi et al. [29] found that maternal preconception nicotine exposure delayed and altered motor development in offspring, using a deep neural network for behavioral classification. Martins et al. [30] developed an AI-based method to standardize the Tail Suspension Test, achieving 95% accuracy in mobility classification using CNNs and machine learning techniques. Wang et al. [31] created a hybrid machine-learning workflow for behavior quantification, using DeepLabCut with random forests, achieving balanced and reliable assessments in various contexts.

### 3.3. Social Behavior Analysis

**Figure 4:**
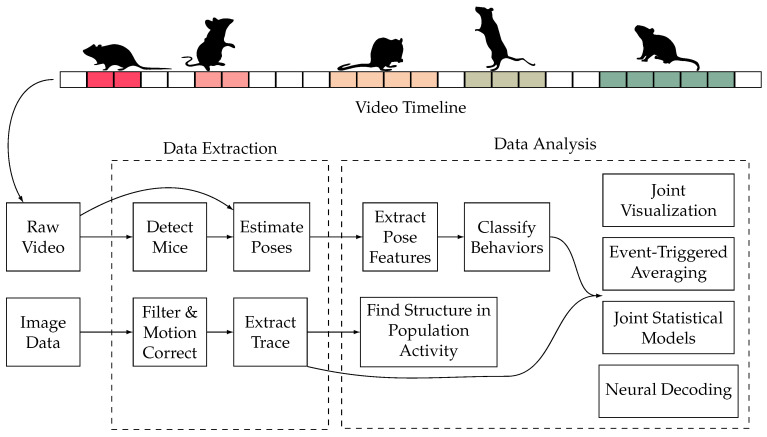
Example of Social Behavior Analysis in Mice.

The study of social behavior in mice [33,34] holds significant importance in medicine. By deeply understanding the neurobiological basis of social behavior in mice, researchers can unravel the mechanisms underlying social behavioral disorders, offering valuable insights for diagnosing and treating related diseases [35,36]. Additionally, research has revealed the impact of social stress and stress on social behavior in mice, highlighting the interaction between stress and social behavior and offering new strategies for treating stress-related disorders. The study of social behavior in mice also contributes to exploring the influence of social interaction on health, providing important clues to understanding the association between social isolation and health issues. Social behavior analysis in mice generally includes object detection, key point detection, post estimation, action recognition, and other tasks, as shown in Figure 4. The process mainly consists of two steps: data extraction and data analysis. In the data extraction step, the researchers estimate the posture of the mice in the video data and extract the mouse trajectories from the image data. In the data analysis step, they extract the pose features and classify behaviors. Finally, the results are visualized.

Current studies of social behavior in mice are vision-based, which means they study the behaviors of mice by analyzing the video data of mice activity. Video data are classified as single-view or multi-view. The studies that rely on analyzing single-view video recordings [37,38] can be ambiguous when the basic information about the behavior is occluded. Multi-view video can provide more behavioral information about mice, making identifying their behavioral characteristics easier. Therefore, multi-view video recordings for observing mice are garnering increasing attention [39,40,41,42].

Segalin et al. [37] introduce the Mouse Action Recognition System (MARS), an automated pipeline for pose estimation and behavior quantification in freely interacting mice. MARS achieves human-level performance in pose estimation and behavior classification, using computer vision and XGBoost, with custom Python code for training novel classifiers. Agbele et al. [38] present a system using local binary patterns and a cascade AdaBoost classifier to detect and classify mice behaviors in videos. This non-invasive method aids animal behaviorists in successfully detecting eight different mouse movements. Winters et al. [43] introduce an automated method using machine learning to assess maternal care in lab mice, improving the reliability and reproducibility of the pup retrieval test. The method achieved 86.7% accuracy in estimating retrieval success, using DeepLabCut for tracking and classification. Jiang et al. [39] propose a multi-view latent-attention and dynamic discriminative model for identifying social behaviors. The model outperforms state-of-the-art methods, handling imbalanced data effectively with a multi-view latent-attention variational autoencoder. Hong et al. [44] develop an integrated system for automatic pose estimation and classification of social behaviors between two mice, enabling rapid measurement and development of new behavioral metrics. Burgos-Artizzu et al. [41] introduce a method for segmenting continuous videos into action “bouts” using a temporal context model. The model combines spatiotemporal energy and trajectory features, achieving a recognition rate of 61.2%, highlighting the temporal context’s importance. Tanas et al. [45] explore multidimensional analysis to evaluate the behavioral phenotype of mice with Angelman syndrome. The approach accurately predicts genotypes and detects treatment improvements using dimensionality reduction and clustering techniques.

### 3.4. Neurobehavioral Assessment

Neurology is a major research direction of biology and medical science. Traditional approaches measure the representational mice behavior information for the neurologic study, such as dynamic weight-bearing test [46], metabolic parameters test [47] and grip strength test [48]. However, some micro features of mice behaviors can promote neurology research, which manual observation cannot discover. Therefore, researchers apply AI methods to analyze certain mice behaviors further to study mice’s nervous systems. To make the neurobehavioral assessment, researchers commonly collect the video of mice behavior, transfer the video data into image frames, and make AI models to train the images for classification, segmentation, key point detection, and context action prediction.

In the neurobehavioral assessment, all the studies collect video data and divide videos into image frames to train AI models. Ren et al. [49], Jiang et al. [50], and Tong et al. [51] collect mice action behavior videos. Geuther et al. [11] collect the mice sleep behavior videos. Cai et al. [52] record the mice freezing behavior videos. Jhuang et al. [53] provide software and an extensive manually annotated video database for data training and testing purposes. Lara-Dona et al. [54] collect videos capturing the pupil behavior of mice in both eyes.

Ren et al. [49] find that automated annotation of mice behavior aids in neuroscience research on long-term memory, using a fine-tuned CNN pre-trained on ImageNet for per-frame image classification. This approach reduced annotation costs and provided more accurate results than other methods. Cai et al. [52] used pre-trained ResNet to study dopamine neuron mechanisms by observing mice freezing behavior, eliminating the need for human scoring. Their model classified behaviors based on pixel intensity differences, achieving 92-96% accuracy within 50 epochs. Jhuang et al. [53] enhanced neurobehavioral analysis by classifying video frames using semantic segmentation and image classification, addressing ambiguous frames. They achieved 93% accuracy with a multi-class SVM, outperforming other systems. Lara-Dona et al. [54] analyzed pupil diameter changes using SOLOv2 for segmentation, reflecting neural activity in the locus coeruleus, and confirmed the system’s high accuracy for real-time tracking. Geuther et al. [11] analyzed sleep quality by video segmentation and EEG/EMG data to train a visual classifier for action recognition, achieving an accuracy of 0.92 ± 0.05, potentially replacing manual classification. Using a CNN network, Tong et al. [51] applied segmentation and key point detection to evaluate the visual function and the nervous system. The system achieved a 94.89% recognition rate by detecting the mouse’s nose position and head orientation. Jiang et al. [50] proposed a hybrid deep learning architecture using a novel hidden Markov model to describe the temporal characteristics of mice behaviors. The system achieved an average accuracy of 96.5% with its combination of unsupervised and supervised layers.

### 3.5. AI Tasks Taxonomy

After summarizing the behavior analysis applications in mice, we also summarized the AI tasks during behavior analysis in mice, as shown in Table 1. The table also summarizes the data types and characteristics of the study.

We classified the literature on mouse behavior analysis applications. We found that most studies can be divided into four categories: disease detection (4 articles), external stimulus assessment (8 articles), social behavior analysis (6 articles), and neurobehavioral assessment (7 articles). In addition, AI-enabled mouse behavior applications can be divided into nine AI tasks based on different research methods. The video data analyzed in most studies are divided into single-view and multi-view, depending on whether the data is collected by one camera or multiple cameras.

## 4. AI-Empowered Approaches

In this section, we focus on the techniques behind mice behavior analysis in biology fields. We first build an AI pyramid according to the dependency relationship between the AI tasks. Then, we introduce several general backbones, namely the fundamental architectures of AI models. Lastly, we introduce the AI models for each AI task. Note that, except for some models used to couple with mice, video data are introduced, and we also introduce some state-of-the-art approaches used for human-related recognition.

### 4.1. AI Pyramid

**Figure 5:**
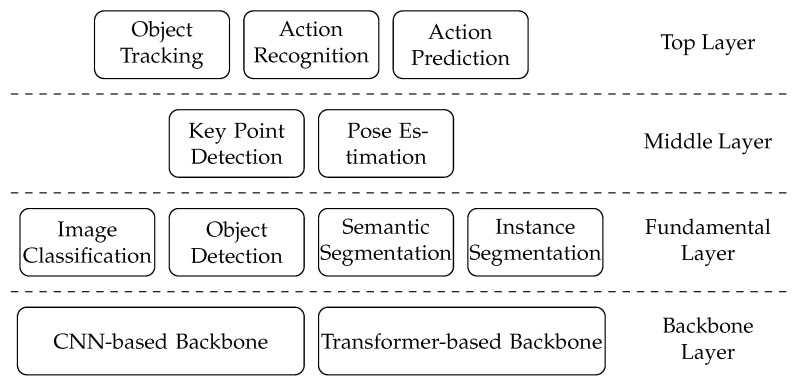
Architecture and organization of artificial intelligence tasks in mouse behavior analysis process.

The architecture of AI tasks is organized as Figure 5. It is a “pyramid” structure that includes four layers: top layer, middle layer, fundamental layer, and backbone layer. The topper layers may take advantage of the techniques of the lower layers.

The backbone layer contains the backbone models and networks. The backbone is the major network of a model. It helps abstract the features of images or videos and generate the feature map for the following network structure. Researchers primarily utilize a pre-trained backbone model and fine-tune it to suit their specific study requirements. The common backbones include CNNs, such as ResNet, ResNeXt, DarkNet, MobileNet, HourGlass, and Transformers.

The fundamental layer encompasses essential AI tasks such as image classification, object detection, semantic segmentation, and instance segmentation. These tasks are atomic and can not be further divided into other AI tasks and take advantage of backbone networks from the backbone layer. For example, object detection can select YoloV5 as the backbone network.

The middle layer contains key point detection and pose estimation. Both tasks may need support from the fundamental layer. For example, the key point detection model may combine semantic and instance segmentation as the first step and apply object detection as the final step. Also, the tasks of the middle layer may apply to the backbone networks from the backbone layer, such as DarkNet and MobileNet.

The top layer tasks may integrate the model’s middle and fundamental layers’ tasks. For example, the action prediction can combine the key point detection (Middle layer) and the semantic segmentation (Fundamental layer) tasks. The task of the top layer can also integrate the backbone network into its model.

### 4.2. Backbone

The backbone is the major architecture of the AI models. It helps extract the modular structure of image features and transform them into high-dimensional feature representations. Existing backbones for mice behavior analysis can be categorized into two main types: CNN-based and Transformer-based architectures.

**Figure 6:**
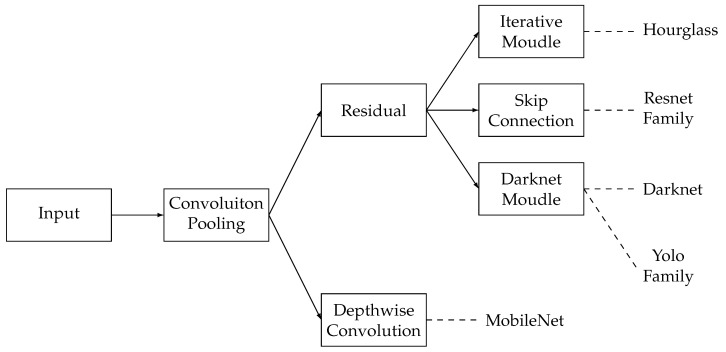
CNN-based Backbone Overview.

In CNN-based backbones, the architecture comprises multiple convolutional layers and pooling layers. The convolutional layers extract features from images, enabling the model to learn and identify patterns effectively. The pooling layers reduce the number of parameters and improve the robustness of features. Common CNN-based backbones include DarkNet, ResNet, MobileNet, and HourGlass. The dependency and relationship of these CNN-based backbones are shown in Figure 6. The CNN-based backbones are all based on convolutions and poolings. In detail, the MobileNet requires depthwise convolution to achieve lightweight, and others require residual techniques to improve performance. In the residual techniques, the HourGlass, ResNet family, and DarkNet family can be categorized by the iterative module, skip connection, and darknet module, which can be further divided into DarkNet.

DarkNet [55] is a lightweight CNN network designed for efficient image processing. Its backbone consists of multiple convolution layers and downsampling layers, with each convolution layer followed by a Batch Normalization layer and a Leaky ReLU layer. DarkNet is well-regarded for its fast detection speed and high accuracy. It is particularly suitable for real-time monitoring of mouse movement trajectories and sudden behaviors, especially in scenarios that require quick responses. However, its ability to recognize complex behaviors, such as subtle social interactions or emotional states, may not be as robust as that of deeper networks.

ResNet50 [56] is a notable example in the ResNet family, characterized as a deep residual network with 50 layers. It effectively addresses the issue of vanishing gradients through residual connections. ResNet’s residual connections enable the training of very deep networks, making it adept at extracting complex features and recognizing intricate patterns in mouse behaviors, such as social interactions and emotional states. However, due to its substantial computational requirements, there may be better choices for real-time applications, particularly in environments with limited resources.

MobileNet [57] is a lightweight convolutional neural network developed by Google, designed for efficient image classification and object detection on mobile and resource-constrained devices. MobileNet’s depthwise separable convolutions significantly reduce the parameters and computational costs, enabling rapid processing and real-time performance on mobile devices. MobileNet is known for its efficient computation and low power consumption, making it suitable for resource-limited environments and allowing for real-time monitoring in laboratory or field settings. However, its accuracy in recognizing subtle behavior changes may not match ResNet’s.

HourGlass [58] is a CNN-based backbone for human pose estimation. It consists of 4 HourGlass modules. Each module contains an input layer, a convolutional layer (64 filters, 7×7 kernel size, stride 2), some residual blocks (64 filters, 3×3 kernel size), a max pooling layer (2×2 kernel size, stride 2), an Hourglass (recursive), some residual blocks (128 filters, 3×3 kernel size), an upsampling layer (2×2 kernel size, nearest-neighbor interpolation), some residual blocks (64 filters, 3×3 kernel size), a convolutional layer (specific filters, 1×1 kernel size), and an output layer. HourGlass networks are designed for pose estimation and keypoint detection and can accurately capture mouse postures and subtle movements, making them ideal for high-precision behavior analysis tasks. However, despite their efficiency, MobileNet’s computational complexity can still be relatively high, potentially requiring significant computational resources. This may make them less suitable for real-time applications, particularly in environments where computational power is limited.

Transformer-based backbone networks have already been applied in mice behavior analysis. Their advanced performance and accuracy make them particularly important in biology. In mice behavior analysis, the encoder of Transformers is used as a backbone network, such as in ViT [59] and Swin [60]. The advantages of applying Transformers include their powerful feature extraction capabilities and efficient recognition of complex behavior patterns. Due to the temporal processing capabilities of Transformers, they can better model animal behavior over time. However, their drawbacks should be noticed, such as the high demand for computational resources and the complexity of model training. These factors limit their widespread application in research environments with limited resources.

ViT was proposed by Google Brain in 2020. It aims to apply a transformer to the computer vision field. ViT contains four layers: the patch embedding layer, transformer encoder layer, global average pooling layer, and the full connection layer. The patch embedding layer divides the image into fixed-sized pieces and maps them into a vector. The transformer encoder layers help to extract the features of the vector. The feature and output presentations use the global average pooling and full connection layers.

Swin was proposed by Microsoft Research Asia in 2021. It has three parts: Swin transformer block for extracting the local feature, stage segmentation for dividing the image into multiple sub-figures, and the cross-stage connection for transmitting the features among different parts.

### 4.3. Fundamental Layer Tasks

The fundamental layer tasks mainly make basic image analysis. These tasks aim to extract information about objects or features from images or videos, such as their location, size, shape, and category.

#### 4.3.1. Image Classification

Image classification is a fundamental task in computer vision, aiming to label an input image with predefined categories. Training approaches include supervised, unsupervised, semi-supervised, self-supervised, and weakly supervised learning. Supervised learning involves learning to map between data and labels using labeled data. Unsupervised learning deals with completely unlabeled data to learn patterns. Semi-supervised learning uses datasets containing labeled and unlabeled data, leveraging labeled data to guide learning while using unlabeled data to enhance performance and generalization. Self-supervised learning also involves learning from unlabeled data, which can be labeled through learning.

Currently, mouse image classification primarily relies on supervised learning. Notably, labeling data requires significant resources, and there is much-unlabeled data in real life. While supervised learning is the predominant method, alternative approaches are valuable, especially when labeled data is scarce, or labeling is costly. Most popular image classification methods now combine supervised and unsupervised learning. The following introduces current advanced image classification algorithms. The summary of image classification is shown in Table 2.

Cai et al. [52] employed supervised learning in their research, using a CNN model to analyze freezing behavior in mice. The CNN was initialized with a pre-trained ResNet18 architecture and further trained on “difference images”, representing pixel intensity differences between consecutive frames, to capture motion. Each difference image was manually labeled as 1 or 0 for “freeze” or “no freeze”, enabling the network to predict labels for new images. This CNN allowed accurate and automated classification of freezing behavior, minimizing manual effort and helping ascertain the temporal relationship between dopamine neuron activity and freezing behavior depending on the VTA subregion. Du et al. [61] introduced an innovative semi-supervised contrastive learning method for classifying esophageal diseases. Using a pre-trained ResNet50 as the CNN backbone, they employed contrastive pair generation and unsupervised contrastive learning to extract features from esophageal gastroscopic images, achieving an accuracy of 92.57%. This method surpassed other semi-supervised methods in accuracy by 2.28% over transfer learning-based methods, reducing reliance on large labeled datasets. Xue et al. [62] crafted a generative self-supervised pretraining approach for few-shot classification in multimodal remote sensing data. Utilizing a transformer structure, images were split and partially masked during pretraining to extract high-level features. Post-pretraining features were combined with spectral data and fed into a lightweight SVM, achieving an Overall Accuracy of 69.63%, an Average Accuracy of 63.15%, and a kappa coefficient of 0.63. Li et al. [63] developed a self-supervised learning framework for diagnosing retinal diseases, using ResNet18 to extract visual features from unlabeled images. Experiments showed that this method outperformed supervised baselines for pathologic myopia and performed comparably for age-related macular degeneration. On the Ichallenge-AMD dataset, the model achieved an AUC of 75.64, an accuracy of 87.09%, a precision of 83.96%, a recall of 75.64%, and an F1-score of 78.51. Taleb et al. [64] explored self-supervised learning techniques for classifying dental caries using unlabeled data. They trained CNNs on 38K unlabeled bitewing radiographs for tooth-level caries classification. Results indicated enhanced performance and improved label efficiency, achieving a sensitivity of 45% with just 18 annotations, comparable to human-level diagnostic performance.

In image classification, supervised, semi-supervised, and self-supervised learning have advantages and challenges. Supervised learning is known for its high accuracy due to its reliance on large labeled datasets. However, labeled data is often scarce in mouse behavior analysis, requiring experimenters’ involvement, thus increasing cost and time. Additionally, supervised learning can easily overfit with insufficient data. Semi-supervised learning combines a small amount of labeled data with many unlabeled data, reducing labeling costs and enhancing generalization but increasing model design and training complexity. The quality of labeled data is crucial as it directly affects performance. Self-supervised learning does not rely on labeled data, learning features through pre-training tasks, making it versatile for extremely scarce labeled data. However, training can be complex, and performance on specific tasks may differ from supervised learning.

**Table 2:** Summary of Studies on Image Classification.

Architecture	Type	Category	Dataset	Performance	Reference
AlexNet, C3D	Mice	Supervised learning	Private	The model can achieve an accuracy of 93.17% on Object Location Memory(OLM) and 95.34% on Novel Object Recognition Memory(NOR).	[49]
ResNet18	Mice	Supervised learning	Private	The classification accuracy of this method can reach 92–96%, with a 5–10% false positive rate(FPR) and 4–6% false negative rate(FNR).	[52]
ResNet50	Stomach	Semi-supervised learning	Private, Kvasir [65]	The classification accuracy stands at 92.57%, surpassing other state-of-the-art semi-supervised methods and exceeding the transfer learning-based classification method by 2.28%.	[61]
Transformer	Remote sensing	Self-supervised learning	Private	The S2FL model shows an Overall Accuracy(OA) of 69.63%, an Average Accuracy(AA) of 63.15%, and a kappa coefficient(κ) of 0.63.	[62]
ResNet18	Retina	Self-supervised learning	Ichallenge-AMD dataset [66], Ichallenge-PM dataset [67]	The model achieved AUC of 75.64, Accuracy of 87.09%, Precision of 83.96%, Recall of 75.64%, and F1-score of 78.51 on the Ichallenge-AMD Dataset.	[63]
ResNet18	Dental caries	Self-supervised learning	Private	With just 18 annotations, the method can achieve a sensitivity of 45%, which is on par with human-level diagnostic performance.	[64]

In mouse behavior analysis, the choice of method depends on data availability and specific needs. Supervised learning is suitable with ample labeled data, semi-supervised learning offers a compromise with limited labeled data, and self-supervised learning provides an innovative solution when labeled data is extremely scarce. Researchers can make informed decisions by weighing the pros and cons of these methods.

#### 4.3.2. Object Detection

**Figure 7:**
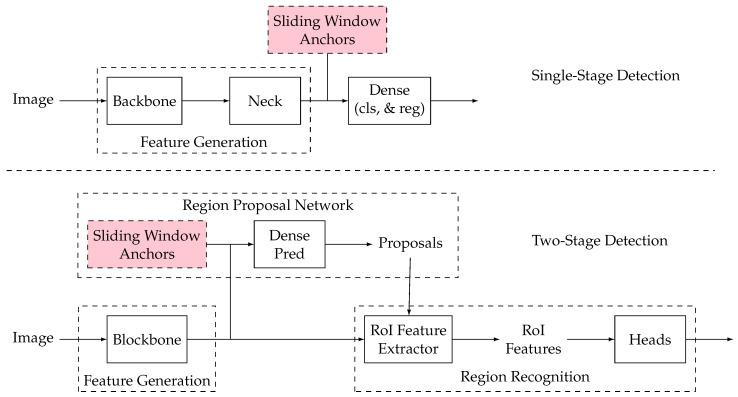
Two different methods for object detection: One-stage and Two-stage Methods.

Object detection addresses the challenge of identifying and locating specific targets. Solutions are generally divided into two categories: one-stage and two-stage methods. Two-stage methods separate the detection task into localization and classification tasks. Initially, a series of candidate boxes are generated using region proposal networks (RPN), followed by classification and regression through the network. Conversely, one-stage methods bypass the RPN by directly regressing the target’s distribution probability and position coordinates, acquiring location information and target categories via the backbone network. The primary processes of these methods are illustrated in Figure 7.

In mice behavior analysis, both one-stage and two-stage methods enhance detection precision. Vidal et al. [26] utilized YoloV3, trained on the Open Images datasets, for one-stage face detection of mice, achieving a mean Intersection over Union (IoU) score of 0.87 after training the model for 100 epochs, with the first 50 epochs freezing all but the output layer. On the other hand, Martins et al. [30] implemented a two-stage method using Inspection ResNetV2 integrated with Faster R-CNN to detect the rear paws of mice, reaching approximately 95% accuracy. Additionally, Segalin et al. [37] applied Inspection ResNetV2 with ImageNet pre-trained weights for locating mice, achieving 0.902 mean average precision (mAP) and 0.924 mean average recall (mAR) in pose estimation metrics on the Behavior Ensemble and Neural Trajectory Observatory (BENTO) dataset. These studies collectively highlight the effectiveness of deep learning techniques in precisely analyzing and detecting various aspects of mice behavior in research settings.

With the advancement of deep learning, state-of-the-art object detection techniques are categorized into anchor-based and anchor-free methods. These methods represent different strategies for detecting and localizing objects within images, each with methodologies and applications. The anchor is used for label allocation. In the anchor-based methods, boxes of different sizes and aspect ratios are preset manually or by clustering methods, which can cover the whole image. It can be applied in both one-stage and two-stage methods. The anchor-free methods can be divided into two sub-methods. The first one determines the object’s center and the predictions for the four borders (called center-based). The second key point-based method involves locating multiple predefined or self-learned key points. These key points are then used to constrain the spatial range of the object, allowing for precise localization and detection within the image. In object detection, anchor-based and anchor-free methods each present unique advantages and challenges. Anchor-based methods detect objects by generating predefined anchor boxes on the image. These methods have developed a mature theoretical framework over the years and can achieve high accuracy on many standard datasets. However, it is highly complex, especially in scenarios requiring real-time processing. Additionally, anchor-based methods may underperform targets that are small in scale or irregular in shape, such as certain postures in mouse behavior, because the predefined anchor boxes might not effectively capture these features.

On the other hand, anchor-free methods achieve detection by directly predicting key points or the center of the target. These methods simplify model design by avoiding the setup of anchor boxes, thus demonstrating greater flexibility in handling targets of varying scales and shapes, particularly suitable for detecting subtle or complex behavioral features. However, the accuracy of anchor-free methods may not match that of finely-tuned anchor-based methods in certain fine-grained detection tasks. Furthermore, since these methods infer key point positions directly from feature maps, they may require more complex feature extraction and discrimination capabilities, potentially leading to a more complex and time-consuming training process. From a fixed camera perspective, anchor-based methods may be more effective in behavior detection scenarios.

In contrast, anchor-free methods have advantages in capturing complex or subtle behavioral changes. Choosing the appropriate method requires balancing specific application scenarios and needs. The state-of-the-art studies on object detection apply the anchor-based and anchor-free modes, summarized in Table 3.

Hu et al. [68] introduce an innovative one-stage anchor-free network that processes point cloud data through voxelization, utilizing the AFDet architecture comprised of convolutional layers and blocks designed for efficient detection, enhanced by self-calibrated convolutions and an IoU-aware confidence scoring mechanism. This system, tested on the Waymo and nuScenes datasets, achieves an accuracy of 73.12% with a latency of 60.06 ms. On the other hand, Li et al. [69] propose a two-stage anchor-based framework utilizing YoloV5 as the backbone, optimized for detecting subtle, similar defects on steel surfaces. This approach integrates advanced preprocessing, feature pyramid, and pixel aggregation networks for robust feature integration and an efficient channel attention mechanism, achieving an mAP of 83.3% on VOC2007 and NEU-DET datasets.

Sun et al. [70] introduce an efficient one-stage video object detection (VOD) system that incorporates a location prior network and a size prior network to address computational efficiency. These enhancements leverage attention mechanisms and feature aggregation from previous frames to improve detection accuracy and speed, achieving 54.1 AP and 60.1 AP. Meanwhile, Zhou et al. [71] present TS4Net, a two-stage anchor-based model specifically designed for rotating object detection. This system builds on the RetinaNet framework, integrating ARM and TS4 mechanisms with an additional IoU prediction head for enhanced accuracy, achieving impressive performance with 96.65 mAP and 98.84% accuracy under the plane category.

Recently, Zhou et al. [72] conducted an advanced study using transformer technology in video object detection. They develop an end-to-end framework employing a spatial-temporal transformer architecture to enhance the efficiency of detection transformers and deformable DETR models. The system uses a ResNet backbone to extract features across multiple frames, followed by shared spatial transformer encoders that generate feature memories, which are then processed by a temporal deformable transformer encoder. A spatial transformer decoder decodes spatial object queries, while a temporal query encoder models relationships between various queries, aggregating support for the current frame’s object query. Temporal object queries and features are fed into the temporal deformable transformer decoder to learn contexts across frames, with video frames serving as input and shared weights forming the output.

#### 4.3.3. Semantic Segmentation

Semantic segmentation is a task in computer vision that involves categorizing each pixel in an image into a defined semantic class. This process allows for a detailed understanding of the image by labeling every pixel according to its corresponding object or region, such as distinguishing between roads, buildings, vehicles, and pedestrians in a street scene. In mice behavior analysis studies, semantic segmentation can be applied to mice behavior video data for behavior recognition and tracking, spatial localization and trajectory analysis, environmental interaction, behavioral context association, disease model, and drug effect evaluation. Semantic segmentation in mice behavior analysis research can achieve fine classification and quantification of behavior, provide more comprehensive and accurate behavioral characterization, and promote a deeper understanding of mouse behavior patterns and biological mechanisms. Current semantic segmentation techniques are categorized into four types based on network architectures, summarized in Table 4.

Vidal et al. [26] introduce a machine-learning technique to automate the prediction of the grimace scale in white-furred mice, a tool used to assess their distress during interventions. This method encompasses face detection, extraction of landmark regions, and expression recognition. It utilizes a unique architecture employing dilated convolutional networks for extracting eye regions and predicting grimace pain. Their approach achieves a performance of 97.2% in terms of accuracy. Dilated convolutional neural networks [73] are also highlighted as powerful tools for performing semantic segmentation.

Wu et al. [74] propose a cutting-edge semantic segmentation framework that excels in segmenting somata and vessels within the mouse brain. The framework includes a CNN for multilabel semantic segmentation, a fusion module that integrates annotated labels with CNN predictions, and a boosting algorithm to iteratively update sample weights. This framework enhances the quality of annotated labels for deep learning-based segmentation tasks. The framework notably enhanced U-Net’s segmentation efficacy, elevating somata accuracy from 0.927 to 0.996 and vessel accuracy from 0.886 to 0.971.

Geuther et al. [11] present a machine learning-based visual classification method for studying sleep in mice, paving the way for high-throughput research. The study involves collecting synchronized high-resolution video and EEG/EMG data from 16 male C57BL/6J mice, extracting temporal and frequency-based features from the video, and training a visual classifier using human expert-scored EEG/EMG data. During video data processing, they employ a segmentation neural network architecture [75] to generate masks for the mice. Their method achieves an overall accuracy of 0.92 ± 0.05 (mean ± SD).

Deep networks capture semantic information in CNN-based architectures, while shallow networks retain rich spatial detail. Zhang et al. [76] propose the EncNet model, which incorporates a context encoding module to capture global semantic information and computes a scaling factor for the feature map to emphasize important information categories. This model was tested on the CIFAR-10 dataset, achieving an error rate of 3.45%. Notable works include the DeepLab family by Chen et al. [77], which reaches 79.7 percent mIOU in semantic segmentation tasks, and the densely connected atrous spatial pyramid pooling (DenseASPP) by Yang et al. [78]. DenseASPP employs dilated convolution to replace traditional down-sampling, expanding the receptive field to gain more contextual information without increasing parameters or computational load. This approach achieved mIoU class of 80.6, iIoU class of 57.9, mIoU category of 90.7, and iIoU category of 78.1 on the Cityscapes test set.

Transformers, based on self-attention, have recently been applied to segmentation tasks. Zheng et al. [79] are pioneers in using transformers for semantic segmentation, creating a segmentation transformer network to extract global semantic data. Their approach achieves impressive results with a mean IoU of 50.28% on the ADE20K dataset, 55.83% mIoU on the Pascal Context dataset, and competitive results on Cityscapes. Inspired by this, Trudel et al. [80] develop Segmenter, a pure transformer model for semantic segmentation. This model leverages pre-trained image classification models, fine-tuning them on moderate-sized datasets for semantic segmentation. Segmenter surpasses previous performance with a mean IoU of 53.63% on the ADE20K dataset.

MLP-based architectures are straightforward, discarding convolution and self-attention. They perform comparably to CNN-based and Transformer-based architectures in various visual tasks. Yu et al. [81] introduce a novel pure MLP architecture, spatial-shift MLP (S2-MLP), which features only channel-mixing MLP. S2-MLP achieves higher recognition accuracy than MLP-mixer when trained on the ImageNet-1K dataset, with the S2-MLP-deep model achieving a top-1 accuracy of 80.7% and a top-5 accuracy of 95.4%.

With their strong local feature extraction capabilities, CNN-based architectures are particularly suitable for analyzing mice’s specific actions or local behavioral features. For instance, when identifying specific movement patterns or local features of mice, CNNs can provide precise detection and classification. However, when analyzing tasks involving global behavior patterns or long-term dependencies, CNNs may not be as effective as other architectures. Transformer architectures demonstrate excellent applicability in mouse behavior analysis, especially when capturing complex global behavior patterns is required. Their self-attention mechanism allows them to handle long-term dependency behavior analysis tasks, such as multi-view behavior analysis or studies involving long-term behavioral changes. However, the application of Transformers may be limited by data volume and computational resources. MLP architectures have relatively limited applicability in mouse behavior analysis and are typically used for tasks where specific features are well-defined or as auxiliary modules in more complex models. Although MLPs are less effective than CNNs and Transformers in capturing complex behavioral features, they can still play a role in specific simple tasks or when combined with other models.

**Table 4:** Comprehensive Review of Semantic Segmentation Studies.

Architecture	Type	Category	Dataset	Performance	Reference
YoloV3	Mice	CNN-based	Open Images dataset	Achieve a performance of 97.2% in terms of accuracy.	[26]
DCNN based on U-Net	Mice	CNN-based	MOST dataset	The framework notably enhanced U-Net’s segmentation efficacy, elevating somata accuracy from 0.927 to 0.996 and vessel accuracy from 0.886 to 0.971.	[74]
-	Mice	-	Private	Achieve an overall accuracy of 0.92 ± 0.05 (mean ± SD).	[11]
Context Encoding Network based on ResNet	Semantic segmentation framework	CNN-based	CIFAR-10 dataset	Achieve an error rate of 3.45%.	[76]
DCNN (VGG-16 or ResNet-101)	Semantic image segmentation model	CNN-based	PASCAL VOC 2012, PASCAL-Context, PASCALPerson-Part, and Cityscapes dataset	Reaching 79.7 percent mIOU.	[77]
DenseASPP, consists of a base network followed by a cascade of atrous convolution layers	Semantic image segmentation in autonomous driving	CNN-based	Cityscapes dataset	DenseASPP achieved mIoU class of 80.6, iIoU class of 57.9, mIoU category of 90.7, and iIoU category of 78.1 on the Cityscapes test set.	[78]
Transformer	Segmentation model	Transformer-based	ADE20K, Pascal Context, and Cityscapes dataset	Achieve new state of the art on ADE20K (50.28% mIoU), Pascal Context (55.83% mIoU) and competitive results on Cityscapes.	[79]
Vision Transformer	Segmentation model	Transformer-based	ADE20K, Pascal Context, and Cityscapes dataset	Achieve a mean IoU of 53.63% on the ADE20K dataset.	[80]
Spatial-shift MLP (S2-MLP), containing only channel-mixing MLPs	Segmentation model	MLP-based	ImageNet-1K dataset	The S2-MLP-deep model achieves a top-1 accuracy of 80.7% and a top-5 accuracy of 95.4%.	[81]

#### 4.3.4. Instance Segmentation

Instance segmentation is a computer vision technique that focuses on finding and outlining each object in an image. The methods of instance segmentation can be divided into three categories: top-down, bottom-up, and one-stage. In mouse behavior studies, instance segmentation can track mouse movement trajectories and postures, allowing for analyzing activity patterns and behavioral characteristics. Instance segmentation provides researchers with accurate and efficient data analysis tools to promote the development and progress of mouse research, the studies of which are summarized in Table 5.

Marks et al. [28] utilize top-down methods and introduce SIPEC: SegNet, a model based on the Mask R-CNN architecture for segmenting instances of animals. They apply transfer learning to the Mask R-CNN ResNet-backbone, pre-trained on the COCO dataset, and apply image augmentation to improve robustness. The experimental results show that SIPEC: SegNet achieves a mean average precision (MAP) of 1.0 ± 0 (mean ± s.e.m.). For videos featuring a single mouse, the model reaches 95% of its peak performance (MAP of 0.95 ± 0.05) with just three labeled frames for training.

Although instance segmentation can play a significant role in mouse behavior recognition, few studies on mouse behavior utilize instance segmentation. The following introduces some popular instance segmentation methods of the above three method categories, which can provide a reference for the subsequent research on mouse behavior.

Shen and colleagues [82] propose a framework for parallel detection and segmentation using only image-level annotations. It combines top-down and bottom-up methods, with a detection component resembling typical weakly supervised object detection designs and a segmentation module using self-supervised learning for extracting class-agnostic foregrounds. Experimental findings indicate that this method surpasses existing benchmarks, achieving cutting-edge results on the PASCAL VOC(49.7% mAP@0.50 score) and COCO datasets(13.1% mAP@0.50 score).

In another study, Korfhage et al. [83] detail a CNN framework based on Mask R-CNN aimed at cell detection and segmentation, which uses nucleus features learned previously for a top-down approach. They implement an innovative fusion of feature pyramids for nucleus and cell detection and segmentation, achieving superior performance on a unique microscopic image dataset created and publicized by the authors, which includes both nucleus and cell signals. The fusion architecture reportedly surpasses current Mask R-CNN methods, offering relative improvements in mean average precision of up to 23.88% for cell detection and 23.17% for segmentation, without utilizing post-processing techniques, to ensure unbiased comparisons.

Zhou et al. [84] propose a bottom-up model for simultaneous learning category-level human semantic segmentation and multi-person pose estimation in an end-to-end manner, using ResNet-101 [56] as the backbone. The model refines a dense-to-sparse projection field and frames joint association as a maximum-weight bipartite matching problem, achieving a differentiable process. Tests conducted on three instance-aware human parsing datasets reveal that this model surpasses various bottom-up alternatives, achieving mAP@0.50 of 39.0% on MHPv2, 49.7% on DensePose-COCO, and 59.0% on PASCAL-Person-Part.

Wang et al. [85] propose SOLO, a ResNet-50-based one-stage, end-to-end instance segmentation framework. The main idea of the SOLO is to transform the instance segmentation problem into a dense prediction problem. The experimental findings revealed that the proposed SOLO framework achieves a mask AP of 39.7% on MS COCO and improves the baseline method by about 1% AP on LVIS, setting new benchmarks in the instance segmentation task and excelling in both speed and accuracy. Additionally, it is significantly more straightforward compared to existing approaches.

Li et al. [86] propose PaFPN-SOLO, an improved SOLO-based instance segmentation algorithm. They enhance the ResNet backbone by incorporating a Non-local operation, effectively preserving more feature information. In addition, they employ a method known as bottom-up path augmentation to extract precise positional information. Tests on COCO2017 and Cityscapes show significant improvements; the average segmentation accuracy on these datasets reaches 56% and 47.3%, respectively, marking an increase of 4.4% and 7.4% over the performance of the original SOLO network.

**Table 5:** Comprehensive Review of Instance Segmentation Studies.

Architecture	Type	Category	Dataset	Performance	Reference
Mask R-CNN	Mice	Top-down method	Private	SIPEC effectively identifies various behaviors of freely moving individual mice and socially interacting non-human primates in a three-dimensional space.	[28]
PDSL framework	-	Top-down method	PASCAL VOC 2012 [87], MS COCO [88]	The PDSL framework surpasses baseline models and attains leading-edge results on both the PASCAL VOC(49.7% mAP@0.50 score) and MS COCO datasets(13.1% mAP@0.50 score).	[82]
Mask R-CNN	Cell	Top-down method	Private	The proposed architecture significantly outperforms a cutting-edge Mask R-CNN method for cell detection and segmentation, delivering relative improvements in mean average precision of up to 23.88% and 23.17%, respectively.	[83]
ResNet101	Human	Bottom-up method	MHPv2 [89], DensePose-COCO [90], PASCAL-Person-Part [91]	Achieve mAP@0.50 of 39.0% on MHPv2, 49.7% on DensePose-COCO, and 59.0% on PASCAL-Person-Part.The proposed model surpasses other bottom-up alternatives, offering significantly more efficient inference.	[84]
ResNet50	-	Top-down method	LVIS [92]	Achieve a mask AP of 39.7% on MS COCO, and improve the baseline method by about 1% AP on LVIS. The proposed framework sets new standards in instance segmentation, excelling in both speed and accuracy, and is notably simpler than current methods.	[85]
ResNet	-	Bottom-up method	COCO2017, Cityscapes [93]	The average segmentation accuracy on COCO2017 and Cityscapes reaches 56% and 47.3% respectively, marking an increase of 4.4% and 7.4% over the performance of the original SOLO network.	[86]

### 4.4. Middle Layer Tasks

The middle-layer tasks mainly focus on pose estimation in humans and other animals. They can be used in motion recognition, human-computer interaction, and motion capture applications. They need higher accuracy and real-time than other tasks to make the estimation more accurate, so they also cost more computing resources than other tasks.

#### 4.4.1. Key Point Detection

Key point detection is a fundamental deep-learning task in computer vision. It serves as a precursor for human action recognition and prediction. In studies focused on mouse behavior analysis, key point detection encompasses essential techniques like object detection and semantic segmentation, taking an image as input and producing key points as output. Typically, key point detection is classified into 2D and 3D detection. In mouse behavior analysis, 2D detection is usually chosen instead of 3D detection. This is because 3D detection requires more complex equipment, such as multiple cameras or depth sensors, which increases the complexity and cost of the equipment and may face limitations in experimental environments. Additionally, processing 3D data demands more computational power without necessarily providing significant improvements in detection outcomes. Therefore, in many cases, 2D detection methods are more practical, efficient, and cost-effective.

Tong et al. [51] focus on identifying key points using semantic segmentation of mouse contours. They introduce a CNN model specifically designed to pinpoint the snout of mice. The architecture includes four convolutional layers, an average pooling layer, a flattened layer, and three fully connected layers. The model inputs a region surrounding the snout and outputs its precise location of the snout point, achieving a recognition rate of 94.89% and a false detection rate of 7.38%.

Wotton et al. [24] develop a CNN based on ResNet50, designed to capture specific features while employing a skipping function to reduce information loss, achieving 98% agreement with human observers per second in small videos. Weber et al. [19], Aljovic et al. [20], and Winters et al. [43] focus on detecting various body parts of mice. They utilize a ResNet-50 model from DeepLabCut, which is trained using 120 frames manually labeled and selected through k-means clustering from multiple videos featuring different mice. The first study identified body parts such as the head, right and left front toes, center front, right and left back toes, center back, and tail base, with an accuracy of 98% when compared baseline to animals at three dpi. The second study detects 14 body part configurations involving both the mother and pup, predicting the injury status with an accuracy of 98% and the recovery status with an accuracy of 94%. The third study labels six body parts (toe, MTP joint, ankle, knee, hip, and iliac crest) across 450 image frames and underwent 400,000 training iterations, with an accuracy of 86.7%.

Besides mice, key point detection is mostly applied in humans. Human key point detection can be categorized into single-person and multi-person detection. The multi-person detection algorithms can be further divided into top-down and bottom-up. All the studies are summarized in Table 6.

Wen et al. [94] use a pre-trained network for object detection and SHNet for multi-person keypoint detection. SHNet consists of four stages and an attention mechanism. The first stage consists of four remaining units, which are composed of a bottleneck with a width of 64. The second, third, and fourth stages contain 1, 4, and 3 communicative blocks. The attention mechanism focuses on channel features with the most information while suppressing unimportant features. The experimental results show that the proposed model Achieves high accuracy on all 16 joint points and has fewer parameters than the previous model.

Gong et al. [95] introduce a retrained AlphaPose model aimed at multi-person key point detection, specifically focusing on the upper human body. The model leverages the regional multiplayer pose estimation (RMPE) framework utilized in AlphaPose, consisting of the symmetric spatial transformation network (SSTN), parametric pose non-maximum suppression (NMS), and the pose-guided proposals generator (PGPG). The SSTN includes the spatial transformation network (STN) for generating human proposals, single-person pose estimation (SPPE) for pose estimation, and the spatial de-transformer network (SDTN) for mapping poses back to the original coordinates. The model can detect 17 key points on the human upper body. This method improves detection precision by 5.6% and reduces the false detection rate in complex backgrounds by 13%, outperforming other state-of-the-art methods while meeting real-time performance requirements.

Zang et al. [96] introduce a lightweight multi-stage attention network (LMANet) tailored for detecting key points on a single person at night. LMANet consists of a backbone network derived from a pruned MobileNet and several subnets that focus on identifying less obvious or hidden key points using different receptive fields and key point associations. The network has been verified on its own labeled dataset, achieving a PCKh score of 83.09%, and on two visible light datasets with PCKh scores of 88.98% and 95.52%, respectively, demonstrating excellent performance.

Hong et al. [97] present PGNet, designed for single-person key-point detection. PGNet is structured around three major components: the Pipeline Guidance Strategy (PGS), Cross-Distance-IoU Loss (CIoU), and Cascaded Fusion Feature Model (CFFM). Utilizing ResNet-50 as the backbone, the model divides it into five stages with CFFM’s aid. A feature-guided network extracts key-point details, while CFFM gathers features from ResNet-50’s conv1-5 layers, minimizing spatial information loss. The method improves the accuracy of the COCO dataset by 0.2%.

#### 4.4.2. Pose Estimation

Quantifying mouse behavior from videos or images is challenging, with pose estimation crucial. While deep learning methods have shown substantial progress in human pose estimation, they face limitations and additional challenges in accurately pinpointing key points when applied directly to mice due to significant physiological differences. Pose estimation for mice can be categorized into two main approaches: 2D and 3D. All the studies are summarized in Table 7.

Zhou et al. [98] introduce GM-SCENet, a novel mouse pose estimation model based on an Hourglass network. The model includes two key modules: the structured context mixer (SCM) and cascaded multi-level supervision (CMLS). The SCM leverages a new graphical model that accounts for motion differences among body parts, while the CMLS provides multi-level information to enhance robustness. By utilizing predictions from both the SCM and CMLS, an inference method is developed to ensure precise localization results. GM-SCENet achieves the state-of-the-art RMSE of 2.84 pixels and the mean PCK score of 97.80%, outperforming other methods on the PCK@0.2 score.

Xu et al. [99] devise a symmetry-based CNN for mouse pose estimation that handles scale variations. It employs a UNet with residual blocks for feature extraction, an Atrous Spatial Pyramid Pooling (ASPP) module to expand the perceptual field, and fuses deep and shallow features to map spatial relationships between body parts. The model predicts the key points using a heatmap and coordinate offset. Using a custom mice dataset, its average PCK was 9%, 6%, and 2% higher than those of CPM, Stacked Hourglass, and DeepLabCut, with superior performance at various thresholds.

Salem et al. [42] propose a method for accurately estimating the 3D pose of mice from single-monocular, fisheye-distorted images using an adapted structured forest algorithm. Their method outperforms existing techniques in continuously predicting mouse behavior from video, achieving a 24.9% failure rate.

In addition to the mentioned studies on mouse pose estimation, we explore advancements in human pose estimation, which hold potential for application to mice pose estimation. Human pose estimation techniques are generally segmented into 2D and 3D pose estimation. 2D human pose estimation aims to track joints and body parts across an image’s surface, while 3D pose estimation aims to determine their positions and depth within the image [100].

2D human pose estimation focuses on accurately localizing human anatomical key points such as elbows and wrists. Sun et al. [101] introduce the High-resolution net (HRNet). This model maintains high-resolution representations throughout the pose estimation process, achieving a 92.3 PCKh@0.5 score and outperforming the stacked hourglass approach. Building on HRNet, Cheng et al. [102] develop HigherHRNet, which uses high-resolution feature pyramids to tackle scale variation in bottom-up multi-person pose estimation. This model achieves 70.5% AP on the COCO test-dev and 67.6% AP on the CrowdPose test, outperforming top-down methods.

Yu et al. [103] propose Lite-HRNet, an efficient high-resolution network incorporating the shuffle block from ShuffleNet into HRNet. Additionally, they introduce a lightweight unit called conditional channel weighting, which replaces the expensive pointwise (1 × 1) convolutions in shuffle blocks—the model achieving superior performance with a PCKh@0.5 score of 87.0, outperforming lightweight networks.

To date, monocular techniques dominate the efforts in 3D pose estimation. However, several innovative approaches for multi-view and whole-body 3D human pose estimation have been proposed. Iskakov et al. [104] propose two solutions for multi-view 3D human pose estimation based on new learnable triangulation methods: one uses confidence-weighted algebraic triangulation, and the other employs volumetric aggregation from 2D feature maps. Their volumetric triangulation reduces MPJPE error by 30%, achieving a mean error of 20.8 mm on Human3.6M. He et al. [105] introduce the “Epipolar Transformer”, which leverages Epipolar constraints for 3D-aware feature extraction, reducing depth ambiguity and achieving MPJPE of 26.9 mm, improving over state-of-the-art by 4.23 mm. Weinzaepfel et al. [106] address whole-body 3D pose detection, combining separate body, hands, and face experts into a unified model using distillation loss. Based on Faster RCNN with ResNet50, this method surpasses similar models without distillation and approaches expert-level performance while being less resource-intensive and capable of real-time operation.

**Table 7:** Comprehensive Review of Pose Estimation Studies.

Architecture	Type	Category	Dataset	Performance	Reference
Hourglass network	Mice	2D	Parkinson’s Disease Mouse Behaviour	Achieve RMSE of 2.84 pixels and the mean PCK score of 97.80%. The superior performance over the other stateof-the-art methods in terms of PCK@0.2 score.	[98]
ResNet, ASPP	Mice	2D	Private	The average PCK was 9%, 6%, and 2% higher than those of CPM, Stacked Hourglass, and DeepLabCut, with superior performance at various thresholds.	[99]
Structured forests	Mice	3D	Private	Achieve a 24.9% failure rate in 3D pose estimation for laboratory mice, outperforming the adapted Cascaded Pose Regression and Deep Neural Networks.	[42]
HRNet	Human	2D	COCO, MPII human pose estimation, and PoseTrack dataset	Achieves a 92.3 PCKh@0.5 score	[101]
HigherHRNet	Human	2D	COCO dataset	The model attains a groundbreaking performance on the COCO test-dev with an AP of 70.5%, and it outperforms all existing top-down approaches on the CrowdPose test, achieving an AP of 67.6%.	[102]
Lite-HRNet	Human	2D	COCO and MPII human pose estimation datasets	Achieves a 87.0 PCKh @0.5 score.	[103]
ResNet-152	Human	3D	Human3.6M and CMU Panoptic datasets	The model reaches cutting-edge performance on the Human3.6M dataset, with a mean error of 20.8 mm.	[104]
ResNet-50	Human	3D	InterHand and Human3.6M datasets	Outperforms state-of-the-art by 4.23mm and achieves MPJPE 26.9 mm	[105]
ResNet-50	Human	3D	MPII, MuPoTs-3D, and RenderedH datasets	The model surpasses the performance of the same whole-body model and remains comparable to that of expert models. It is less resource-intensive than an ensemble of experts and is capable of achieving real-time performance.	[106]

### 4.5. Top Layer Tasks

Top-layer tasks mostly take multiple steps, including those of the lower layer. They are used to analyze and understand the motion in surveillance, robotics, and sports analysis applications.

#### 4.5.1. Object Tracking

Object tracking is the automated method of identifying and following a particular object throughout a sequence of images or video frames. The input of the object tracking algorithm is usually a video sequence, and the output is the information on the target’s position, size, and motion status in different frames of the input video, which is used to achieve continuous tracking of the target. In neuroscience research on mice, object tracking technology can help researchers better understand the mouse’s behavioral patterns and neural activity through monitoring and analyzing the mouse’s behavior. Furthermore, target tracking technology can evaluate mouse behavior performance in drug treatment or nervous system disease models.

Marks et al. [28] introduce SIPEC: SegNet, a Mask R-CNN framework for tracking animal identities. They enhance temporal tracking with SIPEC: IdNet, using a DenseNet backbone and a gated recurrent unit network for reidentification. This approach allows SIPEC to track primates over weeks, outperforming idtracker.ai and PrimNet.

Wang et al. [31] and Winters et al. [43] use DeepLabCut for mouse behavior analysis. DeepLabCut, an open-source markerless pose estimation tool, is based on a ResNet “multi-residual network.” Wang et al. [31] train random forest and hidden Markov models using positional features from DeepLabCut. Conversely, Winters et al. [43] develop a dam-pup tracking algorithm for classifying behaviors like “maternal approach” and “digging”, achieving a notable accuracy of 86.7% for retrieval success, and 99.3%, 98.6%, and 85.0% for “approach”, “carry”, and “digging” behaviors, respectively.

SIPEC: SegNet and DeepLabCut essentially track mice through object detection rather than actual object-tracking models. Many existing object-tracking algorithms for tracking humans and vehicles can be classified into single-branch and multi-branch models. These models can inspire object tracking of mice, as shown in Table 8.

Single-branch models rely on a singular model or algorithm for object tracking, often utilizing linear or nonlinear approaches. Wang et al. [109] propose a framework with a hierarchical single-branch network built on Faster R-CNN [119] with a ResNet-50 [56] backbone. It employs iHOIM loss to integrate detection and reidentification, improving performance in crowded scenes, achieving a MOTA of 50.4% and 51.5% on the MOT16 and MOT20 datasets, respectively.

Multi-branch models leverage multiple models or algorithms for object tracking, often by integrating several linear or nonlinear models to track objects effectively. Vaquero et al. [111] develop a comprehensive detection and tracking system for vehicles in driving environments using a dual-branch CNN architecture. This system employs LiDAR data and a deconvolutional neural network to segment vehicles from a frontal projection. Euclidean clustering is then applied to extract bounding boxes for continuous tracking. The system is further refined by incorporating a dual-view deep-learning pipeline for vehicle segmentation from LiDAR data, innovative techniques such as adaptive threshold recursive clustering, and a bounding box expansion algorithm informed by contextual data. The authors extensively evaluate their approach on the Kitti benchmark [120] for detection and tracking tasks, demonstrating superior performance with values of 39.7% for MOTA, 63.6% for Recall, and 83.0% for Precision, among others.

Jiang et al. [118] introduce a multi-branch and multi-scale perception object tracking framework, MultiBSP, based on Siamese Convolutional Neural Networks. MultiBSP demonstrates robust tracking capabilities and achieves state-of-the-art performance with precision scores of 0.930 and 0.837 for the OTB100 and UAV123 tasks, respectively.

#### 4.5.2. Action Recognition

Action recognition in mice plays a crucial role in biomedical research, as it can be employed for studying disease models, evaluating drug efficacy, investigating the functioning of the nervous system, exploring behavioral genetics, and assessing environmental toxicity. By observing and analyzing the behavioral patterns of mice, insights into disease mechanisms, drug effects, neural network functionality, genetic foundations, and the impact of the environment on organism behavior can be revealed. In this section, we summarize the action recognition-related research; the summarizing results can be referred to in Table 9.

Segalin et al. [37] present the Mouse Action Recognition System (MARS), a suite of tools for automated behavior analysis, including pose estimation and behavior classification. MARS also visualizes neural and behavioral data, accompanied by datasets highlighting inter-annotator variability. The precision and recall of MARS classifiers were comparable to those of human annotators for both attack and close investigation and slightly below human performance for mounting, providing a robust computational pipeline for analyzing social behavior in pairs of interacting mice.

Le et al. [121] propose a framework called LSTM-3DCNN, which utilizes a 3D Convolutional Network (ConvNet) to extract short-term spatiotemporal features from overlapping short video clips. These local features are then input into an LSTM network to learn long-term features that assist in behavior classification. This study demonstrates that the system obtains accuracy that is on par with human assessment.

Kramida et al. [122] develop a mice behavior classification method using LSTM networks. This end-to-end approach extracts visual features with a pre-trained CNN and trains an LSTM model to recognize behaviors. The process employs VGG features and LSTM, producing errors of 3.08%, 14.81%, and 7.4% on the training, validation, and testing sets, respectively.

We also present some state-of-the-art research in human action recognition. Deep learning-based human action recognition methods can be classified as skeleton-based and video-based, depending on whether or not to detect human key points first.

For video-based action recognition methods, most of the network structures are based on Two-stream/Multi-stream 2D CNN [123,124,130], RNN [125,131], and 3D CNN [126,132]. The two-stream 2D CNN framework generally contains two 2D CNN branches taking different input features extracted from RGB videos for HAR, and the final result is usually obtained through fusion strategies. Zong et al. [123] introduce a method called Motion Saliency based multi-stream multiplier ResNets (MSM-ResNets), aiming at enhancing action recognition. Their approach expands the dual-stream CNN model detailed in [130] by incorporating an additional motion saliency stream, thereby improving the capture of critical motion details. In a different approach, Zhang et al. [124] develop two methods for video super-resolution that generate high-resolution outputs, which are then utilized in spatial and temporal streams for action classification. Often, models based on RNNs employ 2D CNNs as feature extractors, subsequently utilizing LSTM models for HAR. Majd et al. [131] advance this concept by proposing the $C^{2}$ LSTM, incorporating elements like convolution and cross-correlation to acquire both motion and spatial features while adeptly managing temporal relationships. Similarly, He et al. [125] implement a bi-directional LSTM framework, combining dual LSTMs to comprehend temporal data in both forward and reverse directions. Moreover, 3D CNN methodologies demonstrate significant efficacy in discerning features pertinent to HAR’s spatial and temporal dimensions. The C3D model [132] effectively extracts spatio-temporal features from unprocessed video content, employing an end-to-end learning strategy. Addressing computational efficiency, Fayyaz et al. [126] tackle the challenge of adapting temporal feature resolution dynamically within 3D CNNs for cost reduction. They propose the SGS module, which equips 3D CNNs with the ability to modify their computational focus by selecting the most salient temporal features, achieving a reduction in computation cost (GFLOPS) between 33% and 53% without compromising accuracy.

For skeleton-based action recognition methods, most of the network structures used in them are based on RNN [127], CNN [128], and GCN [129]. RNNs and their gated variants, such as LSTMs, are proficient in capturing dynamic dependencies in sequential data. Numerous approaches utilize and modify RNNs and LSTMs to adeptly model the temporal context information embedded within skeleton sequences for HAR. Li et al. [127] introduce the Independently Recurrent Neural Network (IndRNN), addressing gradient issues and learning long-term dependencies. Meanwhile, CNNs have demonstrated remarkable success in analyzing 2D images, owing to their exceptional ability to learn features within the spatial domain. Zhang et al. [128] propose view adaptive networks, VA-RNN and VA-CNN, to enhance recognition. Consequently, numerous HAR methods based on Graph Neural Networks (GNN) and Graph Convolutional Networks (GCN) have emerged, treating skeleton data as graph structures with nodes and edges. In this context, Song et al. [129] propose a multi-stream GCN model. This model combines input branches—such as joint positions, motion velocities, and bone features—at an initial phase, employing separable convolutional layers and a compound scaling strategy to significantly minimize redundant trainable parameters while enhancing the model’s capacity, achieving the best performance of the model EfficientGCN-B4 at 92.1% and 96.1% for the X-sub and X-view benchmarks, respectively.

#### 4.5.3. Action Prediction

In some studies, mice behavior needs long-term or short-term observation. The features of mice behavior relate to temporal information. Therefore, the action prediction task needs to relate the context of the former mice’s behavior to predict the future mice’s behavior. Nowadays, temporal context information prediction can be categorized into three parts: short-term temporal context, long-term temporal context, and temporal semantic context. Existing studies of mice context behavior focus on the LSTM models. They also combine the lower layers’ techniques, such as semantic segmentation and key point detection. For example, Kramida et al. [122] use an LSTM model to predict long-term mice behavior, achieving a PCKh value of 92.3 in the MPII dataset and an AP value of 75.5 in the COCO dataset—pre-trained VGG and multimodal background subtraction segment mice from video, integrating semantic segmentation. The LSTM predicts behavior sequences with input, output, and forget gates. Jiang et al. [50] improve the LSTM model of [101] and enhance this with a hidden Markov model for short-term behavior, using key point detection to transform mice interest points into spatial-temporal segment Fisher Vectors for input. Their model achieves a weighted average accuracy of 96.5% using visual and context features and 97.9% when incorporating IDT and TDD features. The model infers hidden states from sequences, explaining dynamics over time.

The state-of-the-art studies on temporal context prediction apply the attention mechanism and transformer framework, increasing the prediction accuracy and efficiency. The studies are summarized in Table 10. For capturing short-term temporal context, Zang et al. [133] introduce a MultiParallel Attention Network (MPAN) to capture contextual information and temporal signals in recommendation systems, targeting users’ short-term preferences. Their approach includes an interest learning module and an interest fusion module. The interest learning module has three components: an embedding layer, a short-term interest generator using a time-aware attention mechanism, and a long-term interest generator utilizing multi-head attention. The interest fusion module applies a bi-linear similarity function for recommendation scoring using the session prefix, outputting a one-hot encoded vector. The MPAN model anticipates users’ short-term interests, achieving Recall@20 of 72.58% and MRR@20 of 33.06% on the YOOCHOOSE dataset and 52.01% and 17.58% on the DIGENTICA dataset.

For long-term temporal context, Guo et al. [134] introduce a transformer-based spatial-temporal graph neural network (ASTGNN) for long-term traffic prediction. ASTGNN features an encoder-decoder architecture with temporal trend-aware self-attention and spatial dynamic GCN blocks. Operating auto-regressively, it uses previously generated data as input for future predictions. A unique self-attention mechanism transforms numerical sequences, capturing temporal dynamics with global receptive fields. It maps queries with key-value pairs to produce outputs as weighted sums. A dynamic graph convolution module captures spatial correlations, integrating self-attention with spatial-temporal encoder-decoder layers comprising temporal trend-aware multi-head self-attention and spatial dynamic GCN blocks. The ASTGNN achieves the best MAE, RMSE, and MAPE values on various PEMS datasets, notably 19.26%, 32.75%, and 8.54% on the PEMS07 dataset.

Zhang et al. [135] introduce MTSCANet with a multi-temporal resolution pyramid structure for temporal action localization. MTSCANet uses a temporal semantic context fusion (TSCF) mechanism to integrate feature sequences of different resolutions into coherent contexts. The local-global attention module (LGAM) encodes input temporal features. Norm and location regularization techniques are applied to produce final results. The TSCF mechanism extracts temporal semantic features, which LGAM encodes for enhanced robustness and enriched content. The model achieves an average mAP of 47.02% on THUMOS14, 34.94% on ActivityNet-1.3, and 28.46% on HACS.

## 5. Discussion

In today’s biomedical research, mice have become an important animal model due to their physiological similarities with humans. With the rapid development of artificial intelligence technology, the application of AI in mouse behavior analysis is also receiving increasing attention. This article aims to explore the application of AI in mouse behavior analysis, emphasizing the potential of AI in identifying and classifying mouse behavior. Traditional methods face difficulties in capturing subtle behavioral features, while AI can automatically extract quantitative features from big datasets, thereby improving the efficiency and accuracy of mouse behavior analysis. The application of AI technology in mouse behavior analysis is becoming increasingly widespread, including disease detection, evaluation of external stimulus effects, social behavior analysis, and neurobehavioral assessment. The selection of AI methods is crucial and must be matched with specific applications. Although AI has shown great potential in mouse behavior analysis, it still faces challenges, such as insufficient datasets and benchmarks. In addition, the study suggests the need for a more integrated AI platform, as well as standardized datasets and benchmarks, to support these analyses and further advance AI-driven mouse behavior analysis. This section will analyze the limitations and challenges of current artificial intelligence technology. In addition, we will discuss the content of future work.

### 5.1. Limitation of AI Technology

The methods for analyzing mouse behavior are mainly derived from human research. However, there are significant differences between human and mouse behavior, and the characteristics of the datasets in these two research fields are also quite different. When these human research and development methods are applied to rodent behavior data, they often need to improve. Firstly, there are areas for improvement in the dataset and benchmarks. Human research can utilize rich and diverse datasets, which provide a solid foundation for training and evaluating AI models. In contrast, datasets for mouse behavior analysis are typically smaller in size and have unique features, making robust training of AI models difficult and limiting the potential for accurate behavior analysis. Therefore, it is urgent to develop standardized datasets and benchmarks that cover the complexity and diversity of rodent behavior to support the development of AI-driven rodent behavior analysis.

Secondly, the applicability of AI models needs to be improved. AI methods for behavior analysis initially developed for human research may not be able to handle specific behavioral characteristics ideally when applied to rodent behavior data. Therefore, it is necessary to develop specialized AI models for mouse behavioral characteristics to enhance their effectiveness and accuracy in this particular environment. Convolutional neural networks (CNNs) perform well in extracting local features, while transformer models have advantages in capturing global behavioral patterns. The hybrid model combining these two has not been fully explored, but its potential is enormous.

Moreover, the lack of a comprehensive testing platform to evaluate AI models’ performance in rodent behavior analysis exacerbates the technological gap. In other fields, such as human behavior analysis, existing benchmarks, and competition platforms are widely used to promote technological competition and innovation. However, such a framework in the field of rodent behavior analysis is needed to compare and evaluate AI models rigorously. Developing a comprehensive testing platform capable of evaluating the complexity of various rodent behaviors is crucial for driving the development of this field.

Finally, the potential of hybrid models has not been fully utilized. Although convolutional neural networks and transformer models have been applied in mouse behavior analysis, the potential of hybrid models that combine both advantages has not been fully explored. More robust and efficient models can be created to handle complex behavior analysis tasks when further developing such hybrid models.

These technological gaps indicate that although AI has shown great potential in mouse behavior analysis, we still need to overcome many challenges in data resources, model applicability, and platform support to achieve more efficient and accurate analysis. Promoting innovation and progress in the fields mentioned above can enhance the application of AI technology in mouse behavior analysis and effectively promote scientific research progress in this field.

### 5.2. Strength and Challenges of Constructing a Mouse Behavior Analysis Platform Framework

A significant advantage of the rodent behavior analysis platform framework is the integration of pre-trained AI models into its database. This integration enables the platform to quickly process various query applications related to rodent behavior analysis, significantly improving analysis efficiency and accuracy. In addition, the platform reduces the threshold for nonprofessionals to use it by allowing them to accept text-based queries, simplifying the query process, and enabling more users to access and utilize the platform’s features easily.

However, despite the significant advantages of rodent behavior analysis platforms, they face some challenges. Firstly, the platform framework requires an effective AI model management module. The rodent behavior analysis platform’s AI model is pre-trained and fine-tuned based on a specific dataset. Over time, the dataset may change, or the model performance may decrease, so it is necessary to update the model parameters regularly. This process needs to be handled with caution to avoid negative impacts on query accuracy. Model updates involve technical adjustments, and it is necessary to consider how to transition seamlessly without affecting system stability. In addition, data migration and compatibility issues during the update process also need to be properly addressed to ensure the continuous and efficient operation of the platform.

Secondly, the platform design requires inter-layer communication, which can lead to an increase in network I/O, thereby affecting the system’s real-time response capability. This network communication overhead may become a performance bottleneck when dealing with many concurrent queries. The platform needs to optimize communication protocols and data transmission methods to reduce latency and improve data transmission efficiency. Meanwhile, it may be necessary to introduce distributed computing and caching mechanisms to share network load and improve overall system performance.

Thirdly, AI models typically require much storage space, challenging the platform’s storage resources. In order to strike a balance between optimizing query performance and minimizing storage requirements, the platform needs to explore methods for compressing models and optimizing storage structures. This may involve using more efficient data storage formats, implementing data deduplication strategies, and utilizing cloud solutions to expand storage capacity. In addition, the platform also needs to consider how to reduce storage space usage without affecting model performance.

Finally, more relevant applications or research on large-scale language models in biology and rodent behavior analysis need to be conducted. This presents new challenges for designing and implementing the query layer of the rodent behavior analysis platform framework. How to apply the advantages of large-scale language models to this field to improve the intelligence level and user experience of the platform is an important direction for future research. Researchers need to explore how to combine natural language processing technology with biological data analysis to develop more intelligent and efficient query systems.

### 5.3. Future Work

This study delves into artificial intelligence techniques for rodent behavior analysis, focusing on applications based on convolutional neural networks and Transformer models. However, the potential of a hybrid model that combines the advantages of these two methods has yet to be fully discussed. Future research could consider developing hybrid models that leverage the advantages of CNN in extracting local features and the ability of Transformer to capture global context, creating more robust models to handle complex behavior analysis tasks.

With the continuous expansion of dataset size in biological research, the potential of hybrid models in processing large-scale data has become particularly important. Researchers can explore how to effectively train and optimize these models to manage larger, more complex datasets. This helps improve the accuracy and efficiency of the model, which performs well in real-time behavior analysis. By optimizing the model architecture and reducing computational complexity, the real-time response capability of the system can be significantly enhanced, which is crucial for applications that require rapid decision-making.

Although this study mainly focuses on rodent behavior analysis, the application of hybrid models can be extended to other biological fields, such as human behavior analysis and ecological monitoring. This will help promote the application of AI technology in a wider range of scientific research. As the complexity of the model increases, ensuring its interpretability and transparency becomes particularly important. Future research should focus on making the decision-making process of hybrid models more transparent so that researchers can understand and trust the model’s output. By exploring these directions, future research can further advance the application and development of AI technology in rodent behavior analysis and other related fields.

In addition, the research results indicate that although AI technology has shown broad prospects in rodent behavior analysis, there is still a significant gap at the technological level compared to human behavior analysis. This gap is mainly reflected in the dataset’s size and diversity, the model’s complexity, and the level of precision in the analysis. Future research should aim to narrow this gap by developing more complex models and more comprehensive datasets to improve the accuracy and reliability of analysis. To this end, establishing standardized datasets and benchmark testing platforms will be a key step, facilitating model training and evaluation and promoting research progress and comparison between different application fields. Through these efforts, the application of AI technology in rodent behavior analysis will become more mature and widespread.

## 6. Conclusions

In this study, we primarily focus on AI-enabled mouse behavior analysis. Firstly, we summarize the applications of AI technology in mouse behavior analysis, including disease detection, evaluation of the effects of external stimuli, social behavior analysis, and neurobehavioral assessment. We then analyze the AI technologies behind these applications and introduce advanced deep learning models related to this field to inspire further research.

During the summarization, we identify some open challenges in AI-enabled mouse behavior analysis research. Firstly, although AI technologies have been widely applied in behavior analysis research, especially in studying human behavior patterns and psychological states, their application in mouse behavior analysis is relatively limited. This restricts the detailed interpretation of mouse behavior and the in-depth analysis of behavior patterns. Secondly, there is a lack of sufficient datasets and benchmarks, and different applications have varying requirements for datasets. This limitation hinders the training and evaluation of AI models, impeding research progress and comparisons across different application areas. Therefore, establishing comprehensive and diverse datasets and benchmarks tailored to specific application needs becomes crucial in advancing AI technology in mouse behavior analysis.

Lastly, an AI testing platform for mouse behavior analysis is currently needed. Although DeepLabCut is a commonly used platform, it only provides the basic steps for mouse behavior analysis and cannot meet the diverse and complex needs of behavior analysis research. Therefore, integrating AI with mouse behavior analysis remains a challenge.

## Figures and Tables

**Table 1:** Artificial Intelligence Task Classification in Mouse Behavior Analysis Process: MV = Multi-view, SV = Single-view; -T = Top-bottom, -B = Bottom-top, -F = Front-Back, -S = Side-Side.

Application	AI Method	AI Task	Data Attribute	Literature
Neurobehavioral Assessment	CNN	Semantic Segmentation, Key Point Detection	SV-T	[51]
	CNN	Image Classification	SV-T	[49]
	CNN	Image Classification	SV-T	[52]
	XGBoost, HMM	Semantic Segmentation, Action Recognition	SV-T	[11]
	SVM,HMM	Semantic Segmentation, Image Classification	SV-F	[53]
	SFV-SAN+HMM	Action Prediction	SV-F	[50]
	SOLOv2	Semantic Segmentation	SV-F	[54]
Social Behavior Analysis	Cascade AdaBoost	Object Detection, Action Recognition	SV-S	[38]
	Random Forest	Pose Estimation, Action Recognition	MV-TFS	[44]
	DeepLabCut	Pose Estimation, Action RecognitionObject Tracing	SV-T	[43]
	MLVAE	Action Recognition, Key Point Detection	MV-TS	[39]
	PCA, K-Means	Action Recognition	MV-TS	[45]
	XGBoost	Object Detection, Pose Estimation, Action recognition	MV-TF	[37]
External Stimuli Effective Assessment	DeepLabCut	Key Point Detection, Action Recognition	MV-B	[24]
	DeepLabCut	Pose Estimation	SV-B	[25]
	YOLO, Dilated CNN	Object Detection, Semantic Segmentation, Image Classification	SV-F	[26]
	PCA, SVM	Action Recognition	SV-T	[27]
	Mask R-CNN	Instance Segmentation, Key Point Detection, Object Tracing, Action Recognition	SV-T	[28]
	CNN, RNN	Action Recognition	SV-T	[29]
	CNN	Object Detection, Action Recognition	SV-F	[30]
	DeepLabCut	Object Tracing, Action Recognition	SV-T	[31]
Disease Detection	CRNN	Semantic Segmentation, Action Recognition	SV-B	[18]
	CRNN	Semantic Segmentation, Action Recognition	SV-T	[16]
	DeepLabCut	Key Point Detection, Pose Estimation	MV-BS	[19]
	DeepLabCut	Key Point Detection, Pose Estimation	SV-S	[20]

**Table 3:** Comprehensive Review of Object Detection Studies.

Architecture	Type	Category	Dataset	Performance	Reference
YoloV3	Mice	One-stage	Open Images dataset	A mean intersection over union (IoU) score of 0.87.	[26]
Inspection ResNetV2 with Faster R-CNN	Mice	Two-stage	Private	Approximately 95%. accuracy	[30]
Inspection ResNetV2 with ImageNet pre-trained weights	Mice	Two-stage	Behavior Ensemble and Neural Trajectory Observatory (BENTO)	0.902 mean average precision(mAP) and 0.924 mean average recall(mAR) in pose estimation metrics.	[37]
Point Cloud Voxelization, 3D Feature Extractor, backbone(AFDet) and the Anchor-Free Detector	Object detection from point clouds	One-stage, anchor-free	Waymo Open Dataset, nuScenes Dataset	Accuracy: 73.12, latency: 60.06 ms.	[68]
YOLOv5, the feature fusion layer, and the multiscale detection layer	Industrial defect detection	Two-stage, anchor-based	VOC2007, NEU-DET, Enriched-NEU-DET	83.3% mAP.	[69]
The location prior network (LPN) and the size prior network (SPN)	Video object detection	One-stage	ImageNet VID	54.1 AP and 60.1 APl.	[70]
ResNet backbone, a FPN, an ARM cascade network with rotated IoU prediction branch, and the two-stage sample selective strategy	Rotating object detection	Two-stage	UAV-ROD	96.65 mAP and 98.84 accuracy under the plane category.	[71]

**Table 6:** Overview of Research on Key Point Detection.

Architecture	Type	Category	Dataset	Performance	Reference
CNN	Mice	2D	Private	Achieve the recognition rate of 94.89% and the false detection rate of 7.38%.	[51]
ResNet-50	Mice	2D	Private	Achieve 98% agreement with human observers per second in small videos.	[24]
ResNet-50	Mice	2D	Private	A 98% accuracy when compared baseline to animals at 3 dpi.	[19]
ResNet-50	Mice	2D	Private	An accuracy of 86.7%	[43]
ResNet-50	Mice	2D	Private	Predict the injury status with 98% accuracy and the recovery status with 94% accuracy.	[20]
SHNet, MaskedNet	Human	multi-person	MPII, COCO2017	Achieves high accuracy on all 16 joint points, comparable to that of the latest models.	[94]
AlphaPose	Human	multi-person	Private, Halpe-FullBody136	Detection precision is improved by 5.6%, and the false detection rate is reduced by 13%	[95]
LMANet	Human	single-person	Private, MPII, AI Challenger	PCKh value is 83.0935	[96]
PGNet	Human	single-person	COCO	Improve the accuracy of the COCO dataset by 0.2%	[97]

**Table 8:** Summary of Studies on Object Tracking.

Architecture	Type	Category	Dataset	Performance	Reference
Mask R-CNN	Mice	-	Private	SIPEC: SegNet is designed to robustly segment animals even in challenging conditions such as occlusions, varying scales, and rapid movements. It facilitates the tracking of animal identities within a session, ensuring accurate and consistent identification despite these complexities.	[28]
ResNet	Mice	-	Private	DeepLabCut can estimate the positions of mouse body parts.	[31]
ResNet	Mice	-	Private	Automated tracking of a dam and a single pup was implemented using DeepLabCut, which was then integrated with automated behavioral classification in Simple Behavioral Analysis (SimBA). Their automated procedure estimated retrieval success with an accuracy of 86.7%, whereas accuracies of “approach”, “carry” and “digging” were estimated at respectively 99.3%, 98.6% and 85.0%.	[43]
Faster R-CNN, ResNet-50	Human	Single-branch	MOT16 [107], MOT20 [108]	Compared to the previous best tracker, it has improved by 1.6%/2.6% MOTA on MOT16/MOT20, Respectively. The specific values are 50.4% and 51.5%.	[109]
DNN	Vehicle	Multi-branch	Kitti [110]	In the Vehicle Tracking task, for MOTA, Recall, Prec. MT, PT, ML The values are 39.7%, 63.6%, 83.0%, 29.5%, 54.6%, and 15.8%, respectively.	[111]
ResNet50	-	Multi-branch	VOT-2018 [112], VOT-2019 [113], OTB-100 [114], UAV123 [115], GOT10k [116], LASOT [117]	MultiBSP demonstrates robust tracking capabilities and achieves state-of-the-art performance. The effectiveness of each module and the overall tracking stability are validated through both qualitative and quantitative analyses.For the OTB100 and UAV123 tasks, the precision scores are 0.930 and 0.837, respectively. On the VOT2018 task, the results of EAO and R were 0.453 and 0.169. For LaSOT, its accuracy is 0.524.	[118]

**Table 9:** Comprehensive Review of Action Recognition Studies.

Architecture	Type	Category	Dataset	Performance	Reference
Hourglass network	Mice	video-based	Private	Provide a robust computational pipeline for the analysis of social behavior in pairs of interacting mice. Precision and Recall of MARS classifiers was comparable to that of human annotators for both attack and close investigation, and slightly below human performance for mounting.	[37]
3D ConvNet, LSTM network	Mice	video-based	Private	Obtain accuracy on par with human assessment	[121]
LSTM	Mice	video-based	Private	Producing errors of 3.08%, 14.81%, and 7.4% on the training, validation, and testing sets respectively	[122]
2D CNN	Human	video-based	UCF101 and HMDB51 datasets	Outperforms other compared state-of-the-art models. The accuracy on the UCF101 dataset is 93.5% and 66.7% on the HMDB51 dataset.	[123]
2D CNN	Human	video-based	UCF101 and HMDB51 datasets	Improve the recognition performance of LR video from 42.81% to 53.59% on spatial stream and from 56.54% to 61.5% on temporal stream.	[124]
RNN	Human	video-based	UCF101 and HMDB51 datasets	The accuracy of the proposed model DB-LSTM on the datasets UCF101 and HMDB51 is 97.3% and 81.2%.	[125]
3D CNN	Human	video-based	Kinetics-600, Kinetics-400, mini-Kinetics, Something-Something V2, UCF101, and HMDB51 datasets	SGS decreases the computation cost (GFLOPS) between 33% and 53% without compromising accuracy.	[126]
RNN	Human	skeleton-based	Penn Treebank (PTB-c), and NTU RGB+D datasets	Performs much better than the traditional RNN, LSTM, and Transformer models on sequential MNIST classification, language modeling, and action recognition tasks. With a deep densely connected IndRNN, the performance is further improved to 84.88% and 90.34% for CS and CV.	[127]
CNN	Human	skeleton-based	NTU RGB+D, the SYSU Human-Object Interaction, the UWA3D, the Northwestern-UCLA, and the SBU Kinect Interaction datasets	Two models were proposed, namely VA-RNN and VA-CNN. The accuracy on NTU-CV is 88.7% and 94.3%, respectively.	[128]
GCN	Human	skeleton-based	NTU RGB+D 60 and 120 datasets	The best performance of the model EfficientGCN-B4 are 92.1% and 96.1% for X-sub and X-view benchmark, respectively.	[129]

**Table 10:** Comprehensive Review of Action Prediction Studies.

Architecture	Type	Category	Dataset	Performance	Reference
RNN with LSTM	Mice	long-term	COCO, MPII	PCKh value is 92.3 in MPII and AP value is 75.5 in COCO	[122]
hidden Markov model (HVV)	Mice	long-term	Private, JHuang’s datasets	Achieve weighted average accuracy of 96.5% (using visual and context features) and 97.9% (incorporated with IDT and TDD features)	[50]
MultiParallel Attention Network (MPAN)	Recommendation	short-term	YOOCHOSE and DIGENTICA	On the YOOCHOOSE dataset, two metrics Recall@20 And MRR@20. The values are: 72.58% and 33.06%. On the DIGENTICA dataset, these two values are: 52.01% and 17.58%.	[133]
Spatial-Temporal Graph Neural Network (ASTGNN)	Traffic forecasting	long-term	Caltrans Performance Measurement System (PeMS)	The best MAE, RMSE, MAPE were obtained on different datasets of PEMS, especially on the PEMS07 dataset, with values of 19.26%, 32.75%, and 8.54%, respectively.	[134]
Multi-temporal resolution pyramid structure model (MTSCANet)	Videos	temporal semantic context	THUMOS14, ActivityNet-1.3, HACS	An average mAP of 47.02% on THUMOS14, an average mAP of 34.94% on ActivityNet-1.3 and an average mAP of 28.46% on HACS	[135]

## Data Availability

Data are contained within the article.

## Abbreviations

The following abbreviations are used in this manuscript.

AI	Artificial Intelligence
CNN	Convolutional Neural Network
RNN	Recurrent Neural Network
CRNN	Convolutional Recurrent Neural Network
FC	Fully Connected
SVMs	Support Vector Machines
KNN	K-nearest Neighbours
MARS	Mouse Action Recognition System
RPN	Region Propose Network
ROI	Region of Interest
IoU	Intersection over Union
GCN	Graph Convolution Network
